# Do we understand children's restlessness? Constructing ecologically valid understandings through reflexive cooperation

**DOI:** 10.3402/qhw.v10.29292

**Published:** 2015-12-21

**Authors:** Anna Helle-Valle, Per-Einar Binder, Brynjulf Stige

**Affiliations:** 1 GAMUT—Grieg Academy Music Therapy Research Centre, The Grieg Academy—Department of Music, University of Bergen, Bergen, Norway; 2 Department of Clinical Psychology, Faculty of Psychology, University of Bergen, Bergen, Norway

**Keywords:** Attention-deficit hyperactivity disorder, cooperative reflexive inquiry, ecological perspectives

## Abstract

Attention-deficit/hyperactivity disorder (ADHD) is the most widely used children's mental health diagnosis today, but the validity of the diagnosis is controversial, for instance, because it might conceal relational and ecological dimensions of restlessness. We invited parents and professionals from one local community in western Norway to participate in cooperative group discussions on how to conceptualize and understand children's restlessness. We carried out a thematic and reflexive analysis of the cooperative group discussions on ADHD and children's restlessness, and present findings related to three ecological levels inspired by Bronfenbrenner's ecological systems model. At the level of the individual, restlessness was discussed as individual trait, as the expectation to be seen and heard, and as a result of traumatization. At the level of dyad, group or family, restlessness was discussed as a relational phenomenon and as parents' problems. At the level of community, restlessness was discussed as lack of cooperation and lack of structures or resources. Our findings show how contextualized and cooperative reflexivity can contribute to more valid understandings of children's restlessness, and how cooperative inquiry can stimulate reflections about solidarity and sustainability in relation to adult's actions.

How do we understand children's restlessness? To what degree is it relevant and ethically defendable to focus on problematic aspects of the individual child's behavior in a de-contextualized manner? What are the consequences of seeing restlessness as an ecologically complex phenomenon? These questions are difficult to answer and might yield a new set of discussions rather than one satisfying answer. A place to start looking for answers, however, is to investigate and challenge current mainstream research and practice in reflexive cooperation with parents and professionals. Attention-deficit/hyperactivity disorder (ADHD) is a much-used concept in both lay and professional language, and can be observed in children's descriptions of themselves, in family life, schools, and mental health institutions, in political and legal documents, and as a structuring concept for research. To shed light on the ecological complexity of children's restlessness, we invited parents and a varied group of professionals from one local community in western Norway to participate in multidisciplinary cooperative group discussions on the topic of children's restlessness.

The ubiquity of the ADHD diagnosis does not automatically prove its ecological and ethical validity, or contribute to sustainable practice. Research on ADHD suggests that it is a reliable concept, but the validity of the diagnosis is still under debate. To contextualize our concerns and research interests, we will now present and discuss a broad, but distilled selection of research on ADHD. This broad overview will be followed by brief reflection on the ecology of human development, before we present findings from a reflexive cooperative group discussion about how to describe and understand children's restlessness in a given context of time and place.

## ADHD as neurobiological disorder

ADHD is described as a neurodevelopmental disorder with a persistent pattern of inattention and/or hyperactivity–impulsivity that interferes with functioning or development (American Psychatric Association, 2013). Inattention, hyperactivity, and impulsivity are exemplified with behaviors like wandering off task, lacking persistence, excessive motor activity when it is not appropriate, talkativeness, or as hasty actions that occur in the moment without forethought and might be harmful to the individual. Manifestations must be present in more than one setting, but signs of the disorder are said to be minimal when the child is receiving frequent rewards, is engaged in especially interesting activities, or is interacting one-on-one.

ADHD is currently the most prevalent psychiatric diagnosis in the child population (Rowland, Lesesne, & Abramowitz, 2002; Ullebø, 2010), with a world-wide pooled prevalence of 5.29% (Polanczyk, De Lima, Horta, Biederman, & Rohde, 2007). Neurological testing has revealed differences between children with and without ADHD in two domains: executive function and motivation. However, neither of these are specific to ADHD (Tripp & Wickens, 2009). ADHD is associated with altered reinforcement sensitivity, but there is a lack of studies that focus on explaining underlying cognitive and neural mechanisms (Luman, Tripp, & Scheres, 2010). Also interesting is that 90% of adults diagnosed with ADHD lack a history of childhood ADHD, nor do they show tested neuropsychological deficits in childhood or adulthood (Moffitt et al., 2015).

### Stimulant treatment of ADHD

In Norway, treatment numbers doubled between 2004 and 2008 from around 12,000 to almost 23,000 individuals (Lillemoen, Kjosavik, Hunskår, & Ruths, 2012). More boys than girls were medicated, and more Norwegian children were prescribed medications than in Finland, Denmark, and Sweden, and fewer than in Iceland. In the UK, the prescription of stimulants to children, adolescents, and adults increased to 7000 prescriptions between 1994 and 2004, from around 6000 to over 450,000 prescriptions (Timimi & Leo, 2009). In 1996, over 11 million prescriptions of Ritalin were written in the United States, with over 6% of all boys taking prescribed stimulants (Timimi & Leo, 2009).

Interviews with children, parents, and professionals show that children's descriptions and experiences of being medicated tend to be more heterogeneous and critical than parents. Children also describe changes in sense of self, adverse effects, and desire to discontinue use of medication (Charach, Yeung, Volpe, Goodale, & Dos Reis, 2014; Olsvold, 2012). Some children report that stimulants improve their capacity for moral agency and increase their ability to meet normative expectations (Singh, 2013).

### ADHD as the mother's project

Fathers tend to be more skeptical than mothers in the face of a possible ADHD diagnosis and medication, but are in general largely absent from research and clinical settings in this field (Singh, 2003). The process of giving a child, often a boy, an ADHD diagnosis and medication is often seen as the mother's project (Olsvold, 2012). The medicalization of children's restlessness can be related to a need to understand and be released from responsibility and guilt (Helle-Valle, 2014; Neufeld & Foy, 2006), but medicalization of children's problem behavior seems to reconstitute oppressive cultural mothering ideals rather than pierce them (Singh, 2004). The physiologically focused explanations for (often boy's) difficult behaviors seem to transfer the blame from mother to brain and facilitate what Singh calls a “no-fault” model of behavior, as organic causes are not morally accountable. Ritalin plays a central part in this absolution of blame, and both mothers and fathers describe how medicating their son with Ritalin reduces the mother's anxiety and contributes to a more pleasant family life. Singh suggests that the medical-scientific enterprise surrounding the ADHD diagnosis is partly dependent on mother's low feelings of self-worth.

Medication might contribute to a more pleasant family life, but for children that are seen as displaying difficult or oppositional behavior, type or intensity of early treatment does not predict functioning 6–8 years later. This being said, children with behavioral and socio-demographic advantage have the best long-term prognosis (Molina et al., 2009). Lower socio-economic status is associated with an overall increased risk of receiving a mental health diagnosis (Bøe, Øverland, Lundervold, & Hysing, 2012), and a correlation between socio-economic status and ADHD seems to be mediated by parent attachment and family conflict (Bøe, 2013; Russell, Ford, Rosenberg, & Kelly, 2014).

### ADHD and violence

A seemingly different but related context is the prevalence of family violence and child abuse. Maltreated children typically struggle with regulating affect, attention, and social bonds, and ADHD is a common diagnose in this population (Ackerman, Newton, McPherson, Jones, & Dykman, 1998; Van der Kolk, 2005). There is also a strong link between childhood abuse and adult ADD or ADHD (Fuller-Thomson, Mehta, & Valeo, 2014). In the Nordic countries at least 3–9% of the child population experience severe physical abuse, and at least 7–12.5% witness violence in the family (Kloppen, Mæhle, Kvello, Haugland, & Breivik, 2014). Global prevalence of child maltreatment is estimated to be 12.7% for sexual abuse, 22.6% for physical abuse, 36.3% for emotional abuse, 16.3% for physical neglect, and 18.4% for emotional neglect (Stoltenborgh, Bakermans-Kranenburg, Alink, & Van Ijzendoorn, 2015). These numbers clearly state that child maltreatment is a huge global problem and a significant threat to the health and well-being of children that currently are not integrated in the discussions about children's restlessness that focus on their behaviors. In Norway, however, the former Minister of Children, Equality and Social Inclusion recently authored a book in which she argues that violence against children best can be described as a national taboo, and that the ADHD diagnosis plays an important role in “not seeing” the most vulnerable children (Thorkildsen, 2015).

### An ecological systems approach to children's restlessness

One way to understand ADHD is to summarize it as an individual's problem that is connected to, as well as manifests itself on a biological, psychological, and social level. The research presented in this introduction confirms that children that fit an ADHD diagnosis struggle on many levels including academic performance, motivation, and in relation to parents and peers. This could indicate that the problems stem from the children themselves. On the other hand, ADHD can also be related to adverse childhood experiences, parental attachment, and socio-economic status. These findings highlight the importance of context, for instance, familial or societal conditions for children's attachment and play (Navarez, Panksepp, Schore, & Gleason, 2013; Panksepp, 1998).

That child development is a function of the ecological systems that they are a part of, and is a central premise in Bronfenbrenner's (1977, 1979) ecological system's model. The ecological environment is understood as a set of nested structures. The innermost level (micro level) is the immediate setting of the developing child, like the home or kindergarten. The child's development is affected not only by experiences in these immediate settings, but also by the relations between these settings (meso level) and by events occurring in settings where the child is not even present, like overarching patterns of ideology and organizations of social institutions (macro level). Bronfenbrenner's conceptualizations have later been elaborated and reformulated, for instance, in community psychology, where Dalton, Elias, and Wandersman (2007) have suggested a revision of the model, with the person in the center, surrounded by ecological layers of microsystems, organizations, localities, and macrosystems.

To study restlessness not only as a function of children's immediate settings but also in relation to overarching ideologies and structures, we have chosen to use an ecological systems approach in our analysis. In this article, we address the problem: How do parents and different professionals conceptualize and understand children's restlessness when they are invited to think about and beyond the diagnosis of ADHD?

## Method

We invited participants to join a cooperative inquiry group, with an emphasis on ecological and interdisciplinary reflexivity (Alvesson & Sköldberg, 2000; Singh, Filipe, Bard, Bergey, & Baker, 2013). Cooperative inquiry is a form of action research aimed at strengthening the ecological validity of knowledge, and where research can be looked upon as a form of “lived inquiry” (Heron & Reason, 2001). The research process entails moving between reflection and action in a systematic and increasingly refined way (Hummelvoll, 2006). The cooperative inquiry group was part of a research project on community music therapy in kindergarten. The community music therapy project was carried out in parallel with the cooperative inquiry group discussions and ended with a performance that the participants in the cooperative inquiry group was invited to attend. The thirteen 5-year-old children that attended the community music therapy project attended the kindergarten that also hosted the cooperative inquiry group. The music therapy project will not be further presented or discussed in this article, since music therapy is not specifically addressed in this study.

### Participants and procedure

Participants were formally invited through an information sheet titled, *Music therapy in kindergarten: a different way to meet restlessness?* The research project was presented as an invitation to discuss and expand on current perspectives and practice, and music therapy was introduced as an alternative and resource-oriented approach to restlessness. We contacted local institutions that were involved in assessing and making decisions about children's restlessness and included those willing to participate. For ethical and practical reasons, the kindergarten distributed the information sheet to the parents, and parents were recruited through the kindergarten. As a part of this recruitment procedure, the first author and principal investigator (Helle-Valle) was invited to present the project during a meeting with the parent representatives. The three fathers that were elected as parent representatives in the kindergarten were interested in the project right away and wanted to participate without trying to recruit some of the other parents. Given the general absence of fathers in ADHD research, and because of the high number of female professionals in the group, we were happy to include the three fathers without further recruitment procedures.

The cooperative inquiry group ended up consisting of three men and seven women: the three fathers, two pedagogical leaders and the director of the kindergarten, a music therapist and a psychologist from the pedagogical–psychological services, a caseworker from Child Welfare, a clinical social worker from Child and Adolescent Psychiatry Unit, and a General Practitioner (GP). A music therapist from the pedagogical–psychological services from a different part of town and a researcher with a background in psychology facilitated the group discussions (Helle-Valle).

### Outline of the group meetings

The cooperative inquiry group met four times, with some weeks in between each meeting, so that the first meeting took place in February and the fourth and final group meeting took place 7 months after the first. The discussions were improvised around the topic of children's restlessness based on the agenda of the participants and took the form of an informal and focused discussion. If some participants did not take part in the discussions, the facilitators would try to include them by relating the discussion to their context. The facilitators also shared their point of view and their experience, as this was a collaborative discussion and not a focus group interview. The first meeting was used to introduce the participants to each other and to share information about one's own context and initiate reflections on children's restlessness. The group members were interested in findings and critical perspectives related to ADHD, and the facilitators presented similar perspectives as those given in this introduction. The second meeting consisted of reflections on adults’ actions, the third meeting was used to watch recordings of the community music therapy project and discuss these, and the fourth meeting was used to continue discussions from the two first meetings, as well as spend some time evaluating the research process. All group meetings were hosted by the kindergarten and lasted 2 h, apart from the fourth meeting that was extended by 1 h.

### Ethical considerations

Ethical approval was obtained from the regional committee for medical and health research ethics (protocol: 2013/1281/REK vest). Informed consent procedures rested on written information sheets that were signed by all participants, and verbal information to the kindergarten, the pedagogical–psychological services, and the children's welfare. A verbal agreement in the first group meeting underlined the importance that individual children were not discussed in the group, and that participants were to draw on their own experience or anonymized examples in discussing children and restlessness.

We were able to recruit three fathers to the cooperative inquiry group, but all of the professional participants were women. Despite profound changes related to gender equality and employment in the Nordic societies over the last 50 years, there is still more women than men who work with children. In our cooperative inquiry group, the three parent representatives were men, and this gave us an interesting point of departure for the discussions as fathers’ voices tend to be underrepresented in research on ADHD. During the discussions in the cooperative inquiry group, fathers tended to ask more questions and the professional participants tended to provide answers. This being said, the fathers were very engaged in the process, expressed their concern that ADHD had become such an influential perspective, and wanted to know more about the forces behind this development. One of the fathers, for reasons unknown to the authors of this article, only attended the first meeting. We do not know why this happened, and the reasons might be practical or personal without any connection to the topic discussed. However, it could also be understood in light of Singh's (2004) and Olsvold's (2012) research on ADHD as the mother's project, where fathers’ absence could be interpreted as an avoidant expression of disagreement.

Critical and reflexive research is needed to prompt and inform critical reflection in everyday practice. However, critical research on pathology and power issues in relation to children will most likely touch upon sensitive issues. We have done our best to facilitate the cooperative inquiry group in an informative and truly cooperative manner, to carry out the analysis with the group in mind and through their direct cooperation, and to present and discuss the findings in a reflexive way without compromising the integrity of the participants or of the children they described.

### Data collection

All group meetings were audio-recorded, transcribed verbatim, and anonymized. Care was taken to remove all information that could directly or indirectly identify actual people, children, or places when using extracts from the transcriptions.

### Analysis

We used two complementary and interrelated approaches when analyzing the data: thematic analysis and a reflexive approach. The thematic analysis of the transcribed text was carried out by the first author in a stage-wise process (Binder, Holgersen, & Moltu, 2012; Braun & Clarke, 2012) that is described in further detail below. Before, during, and after the thematic analysis, both the authors and the members of the cooperative inquiry group were involved in an analytical process that can be described as explorative and reflexive (Alvesson & Sköldberg, 2000; Binder et al., 2012). Within this interdisciplinary and open reflexive approach, we analyzed the data on the premise that children's development is an ecologically situated phenomenon (Bronfenbrenner, 1979).

In summary, our analytic process was carried out in several stages: (1) the first author who also facilitated the group discussions noted her reflections after each group discussions and discussed these with her co-facilitator and the co-authors of this article. This created opportunities for reflexive dialogue about the processes in the co-operative inquiry group and also served to update the researchers that had not been part of the group discussions. (2) After each meeting, the first author and facilitator made a summary of the group discussions and emailed these to the participants in the co-operative inquiry group. This was done to help the participants remember what we had discussed or to inform those who had missed the meeting, support dissemination by the participants in their respective practice fields, strengthen the group's identity, and to indirectly remind the participants about our availability for input and comments between meetings. (3) After the last meeting in the cooperative inquiry group, the first author analyzed the transcribed audio recordings for themes that were regarded as relevant to the research question. These were how to understand children's restlessness, how to handle restlessness in practice, power issues related to structure and responsibility, reflections on practice improvement and *status quo* in relation to prevention, health promotion, cooperation, and resources. The original transcribed material counted close to 90,000 words. For the purpose of this article, the authors decided to focus on the participants’ *understandings* of children's restlessness. (4) Themes and codes related to the overarching category *Restlessness understandings* were re-analyzed from an ecological systems perspective. Ecological levels were discussed and adjusted in relation to the themes and codes, and we ended up with three levels slightly different from the micro, meso, exo, and macro levels described by Bronfenbrenner (1979). Our levels more closely correspond to that of Dalton et al. (2007) described above. We defined the first ecological level of analysis as the individual child, the second as dyad, group, or family, and the third as community. Discussions would often include several ecological levels, so we chose to place findings according to the focus that was emphasized. (5) A first draft of the article was sent to all the co-authors and members of the cooperative inquiry group for comments. (6) The first author completed the article informed by these comments.

## Results

*Level of the individual* contains the themes *Restlessness as individual trait*, *Restlessness as expectation to be seen and heard*, and *Restlessness as a result of traumatization*. *Level of dyad, group, or family* contains the themes *Restlessness as relational phenomenon* and *Restlessness as parents’ problems*. *Level of community* contains the themes *Restlessness as lack of cooperation* and *Restlessness as lack of structures or resources*. Restlessness as children's needs to be seen and heard and in relation to traumatization has been placed under *Level of the individual*, despite the fact that these themes imply a relation. We have chosen to place these themes at the *Level of the individual* because participants reflected on the individual child's behavior and what this behavior could communicate.

### Level of the individual

#### Restlessness as individual trait

Children's restless behavior was a focus throughout the discussions, and there were many direct or indirect descriptions of “the problem child.” Cooperative, polite, and generous behavior was seen as desirable and adaptive behaviors that could give the child a sense of mastering. The group also reflected on restlessness as a trait relating to personality, including restlessness as a sign of creative talent. Restlessness was often talked about as externalized, but the psychologist from the pedagogical–psychological service was especially interested in and concerned by children's internalized or “invisible” restlessness.

There was a general concern that ADHD exaggerated the focus on individual symptoms and function at the expense of resources, hope, and ecological complexity. Framing restlessness as ADHD could make the individual passive, promote hopelessness, induce guilt, and shift responsibility from adults and society to individual children. Being restless was talked about as being a problem, but the participant from Child and Adolescent Psychiatry Unit referred from a conversation with a patient: “I talked to a boy the other day, with his family, and then it was like we talked about ‘Some children grow out of it’, and then he said ‘Yes, I hope I don't!’”

The GP reported that discussions in the cooperative inquiry group had made her reflect on the ecological complexity of children's restlessness. Her appraisal was that children's restlessness was often related to family or societal problems, and she described the limitations of using an individual-oriented diagnosis when she experienced the problem as a complex family and health system situation. She reported that the group discussions had led to a decline in referrals regarding ADHD, and an increase in cooperative initiatives. The music therapists and the psychologists in the group challenged the GP to ask parents about the child's resources during consultations and include these in the formal referral documents. The GP went on to suggest that referrals could contain a short description of context for observations and that the GP also could include a short reflection pertaining to the limitations of observing a child in such a setting.

Restlessness was often talked about as externalized, but the psychologist from the pedagogical–psychological service was especially interested in and concerned by children's internalized or “invisible” restlessness. Participants were both concerned about how the individual child was to be understood, but also saw the need to look beyond the individual and acknowledge contextual factors to better understand the child's restless behavior.

#### Restlessness as expectation to be seen and heard

The kindergarten teachers talked about restlessness as something stemming from today's children's expectations of being seen and heard. Today's children were described as more self-centered and less generous than before, and this tendency was seen as especially problematic in group settings. Being seen, heard, and respected was to a certain extent talked about as important in relation to adult needs, and as a relational challenge or effort in relation to children's needs.

At the end of the first meeting, one of the fathers suggested that the group should investigate their own contribution to restlessness in everyday settings. After this, the group shared personal experiences with being restless, for instance, stemming from boredom or a feeling of being invalidated.

#### Restlessness as a result of traumatization

The overlapping qualities of behavior fitting the ADHD diagnostic criteria and behavior stemming from complex traumatization were discussed throughout the meetings. ADHD was discussed as potentially being a sign of family violence, masking the violence, or giving adults excuses to handle their children roughly. ADHD was seen as indirectly facilitating adult displacement or avoidance of responsibility. The participant from Child Welfare commented on the high occurrence of ADHD in the records of children in the Child Welfare system and described an encounter with a now grown up man that had been admitted on several occasions during his childhood. He told her how every emergency hospitalization for psychosis was caused by his stepfather “beating him senseless.”

One of the longest and most charged pauses occurred after a comment about our responsibility as adults and as a community to discover and help children that are exposed to family violence. One of the participants went on to formulate the question “Do we have a good enough understanding of the child?” emphasizing that we should not quit until we do. “Understanding the child” became a recurring topic throughout the four meetings.

### Level of dyad, group, or family

#### Restlessness as a relational phenomenon

Restlessness was often described as existing between children, or between adults and children. Both the kindergarten teachers and the fathers saw restlessness as a way for children to get attention from others when feeling insecure, or as a sign that they did not respect your authority. The participant from the Child and Adolescent Psychiatry Unit talked about the importance of allowing children to express their needs while keeping one's place in the driver's seat. She went on to explain that struggling children only have two choices, withdrawal or restlessness, and that both should be understood as communication.

The kindergarten teachers described how restlessness arose in certain constellations of children, and how it became a problem when there were too many children per adult. The kindergarten director held the ideal ratio to be 4 adults per 16–18 children. An everyday problem in kindergarten was to regulate restlessness in a group of children where some needed more stimulation and others were easily overstimulated. The kindergarten teachers and the music therapists described how restlessness would form in gaps between structured activities, for instance, when children were supposed to stop one activity and start something new. All participants with experience of being pedagogical leaders in kindergarten—the music therapist facilitator, kindergarten teachers, and the kindergarten director—also discussed children's creativity as an everyday challenge and as a relational problem. The fathers were interested in children's ability to act in an open and including way, and to promote a sense of mastering.

#### Restlessness as parents’ problems

The participant from the Child and Adolescent Psychiatry Unit shared what she called a perplexing experience of hearing a group of well-educated, well-to-do mothers all wondering if their child had ADHD. The participants with grown up children described today's parents as more insecure about making decisions. The group discussed how child rearing had changed over the last decades regarding children's participation. Parent insecurity was held to be a negative development, but also the price one had to pay for understanding children better. Modern child rearing was seen as characterized by compromise.

Several participants highlighted the connection between tired adults and reduced tolerance for restlessness. Children's restlessness was often discussed as a sign of parent's problems, and ADHD as a framework for understanding restlessness could be seen as facilitating parents’ lack of awareness or willingness to deal with these problems. The other music therapists described how two of her friends struggled with their relationship, and how despite being on their best behavior, one could “cut the tension with a knife.” She shared her own feeling of being uneasy as if there was a “constant underlying vibration” when spending time with them, and related her experience to children's incapacity to deal with such issues, possibly leading to experiences of shame and self-blame.

The participant from Child and Adolescent Psychiatry said that restlessness does not always need to be referred and examined, but that many parents could benefit from using less negative or critical parenting strategies and rather learn about children's need for support in regulating their feelings and relationships. Professionals should take care to place relational responsibility with the parents, even if it is experienced as challenging.

### Level of community

#### Restlessness as lack of cooperation

The participant from the Child Welfare Unit talked about restlessness in relation to children or families that did not fit in, that were not given relevant help, or any help at all. The kindergarten personnel were satisfied with current cooperation with parents, with the Child Welfare Services, and with an interdisciplinary consultation team in their local community. The GP, however, shared an example of how current cooperation could cause restlessness and how it failed to meet the complex ecological needs of vulnerable children:

There was a [little girl] who had experienced that her mother for the third time this summer was admitted with paranoid psychosis and was really sick. And in this case there was a grandmother around that took care of the [girl] in relation to admission. But then the mother was released from hospital, and the Child Welfare Services were in the picture. The mother had been released and wasn't paranoid any more, but she struggled with her own things. And there was a [small baby] in this, and then the mother turns up with the [little girl] in my office and says “The Child Welfare Services says that I have to refer her to the Child and Adolescent Psychiatry Unit, because she has these tantrums, she is so restless” […]And it is clear what is missing, [it] is follow up from adult psychiatry where the mother had been admitted. She is actually going home to the responsibility of two children—the father was peripheral in this. So I refer to the Child and Adolescent Psychiatric Unit, because I thought that “one has to get involved and do something,” but then the Child and Adolescent Psychiatric Unit makes a sensible assessment that the anger has to do with her life situation. Maybe it was related to the fact that it was the middle of the summer holidays, because there was a psychologist at the Adult Psychiatric Unit that was supposed to follow up the children of those admitted, but it seemed to have slipped, and there wasn't any sort of follow up. And the girl gets an appointment [six months later]! And then the mother reacts to this “It's *now* that we needed- ” But I agree with that assessment, because it's not a diagnosis for the daughter, it is *help* in that life situation. There are some holes, sometimes, where children fall between several chairs.

The GP criticized how current practice too often rests on a clinician's availability, interest, and willingness to spend resources on this. Two approaches within the mental health care system were discussed: one quick with a focus on changing the child that often involves medication from day 1 and another a slower approach with a focus on changing the child's everyday situation by cooperating with adults in the child's immediate context.

#### Restlessness as lack of structures or resources

The restlessness was experienced as particularly challenging when arising in places or ways that challenged the adults’ competence, preferences, architecture, or number of staff. Examples of this were rough and tumble play inside, children with opposite needs sitting next to each other during a meal, or children with suspected complex trauma that the kindergarten worried about, but felt incompetent in helping.

The group discussed processes of negotiating the structural possibilities and limitations in the community. The fathers and kindergarten teachers described how sports were an important social arena, and how practice or summer camp sometimes functioned as a relief for tired parents in spite of their child's interest or talent.

Restlessness was related to the architecture in two different ways. The first was construction of the kindergarten buildings and whether the architecture supported or hindered children's development. The second perspective on architecture was how children often can hear parents arguing through the walls, and how parents sometimes think that as long as they argue after the child has gone to bed, it does not affect the child.

The group discussed how children's creative talents, or creatively gifted children, are met by the different systems and generally understood. Several members expressed concern that children's creativity tends to be systematically misunderstood and overlooked, and rarely used as a resource for change. The music therapists discussed how music could facilitate a playful approach to restlessness, and that music therapists could support kindergarten personnel in this process.

## Discussion

Therefore, how do parents and different professionals conceptualize and understand children's restlessness when they are invited to think about and beyond the diagnosis of ADHD? Our findings show that children's restlessness can be conceptualized as a many-layered ecological phenomenon that spans from the child's problems and resources to restlessness as a relational phenomenon, to resources and structures in the local community, and to overarching perspectives on how children's restlessness can be understood in relation to individual and context. The participants reflected on children's need to be seen and heard not only as important, but also as a relational and cultural challenge. “Do we have a good enough understanding of the child?” ended up being a central question during the discussions and points to a pragmatic aspect of understanding and to the possibility that ADHD might not be a *good enough* understanding in this respect.

Based on our results, we argue that increased reflexivity can contribute to increase the validity of research and everyday understandings of children's restlessness. Furthermore, the process of this cooperative inquiry stimulated participants to reflect on the solidarity and the sustainability of current practice. The participants did not use these terms themselves, but worried about the deflection of responsibility that ADHD seemed to facilitate; a focus on problematic behavior can prevent adults from seeing their own contribution, underlying problems, and contextual factors. In interpreting the material, we choose to use the word *solidarity* to highlight this. Solidarity is one of the central values informing the Universal Declaration of Human Rights, but as Stjernø (2004) clarifies, solidarity is a multifaceted concept. Solidarity might involve attempts of realizing common interests as well as attempts of realizing a better world. During discussions, both dimensions were present, and the participants stressed concerns about being responsible human beings in context. During discussions, participants often pointed to adults’ responsibility, which again led the group to reflect on restlessness as both a co-created and a shared problem. Judging by the reflections in the group, it seems that the sustainability of perspectives and practice depends on such efforts of solidarity. Based on our findings, we also argue for the need to integrate research on ADHD with research on child maltreatment and point to the possible tension between ADHD and a child perspective and children's own perspectives.

### Validity, solidarity, and sustainability

Both current research on ADHD and our findings indicate that adults experience children's restless behaviors as problematic, and that restlessness can be understood as “impaired function” in an everyday setting. The participants in the cooperative inquiry group had many descriptions of “the problem child,” but also sensed the need to look beyond the behavioral problems of the individual child and acknowledge resources and contextual factors. Our results show that when adults who are involved in children's everyday lives reflect on ADHD, they question biomedical explanations and point to the risks of an exaggerated focus on individual pathology. The participants rather understood restlessness as a situated and contextual phenomenon that needs to be approached from a variety of perspectives, including the children's own perspectives. This meant allowing children to express their needs and perspectives, and simultaneously to remind parents about their responsibility to “keep their place in the driver's seat” as it was coined by one of the participants.

The importance of balancing children's freedom of expression with an adult perspective on responsibility and community can be related to ethical perspectives on community and ecological sustainability that point to the need for an increased sense of *firmness* in child rearing and society at large (Foros & Vetlesen, 2012). This need for firmness was discussed indirectly when participants wondered if seeing and hearing children to the extent that is common in Norway today can make them self-centered as well as difficult to handle in groups since children might lack awareness of others and of community on both an individual and societal level. The tension between being seen and heard oneself vs. being aware of others and of community could be understood in light of tension between the Convention on the Rights of the Child (United Nations, 1989), where children's rights to be seen and heard are described, and the descriptions of how children's should not behave (e.g., the ADHD diagnosis). This macro level tension could contribute to both national and international discussions about the frames within which children are raised and understood. Tracing the tension between individual and group from macro to micro could contribute to a better understanding and contextualization of children's restlessness.

Our results show that restlessness can be understood in relation to individual children's problems or creative talent, but also as *contexts* that impair children's function and create symptoms of restlessness. It is interesting that the participants in this cooperative inquiry group resisted the biomedical perspective of ADHD and the medicalization of restlessness despite its immense influence. Instead, parents, teachers, therapists as well as the GP constantly returned to relational processes, cooperation, and the need for a deeper and contextualized understanding. Rather than highlighting the need for efficiency and reliability, two central strengths of an ADHD approach, our findings point to the need for increased validity, solidarity with children's problems as they experience them, and to focus on the sustainability of change by looking beyond the perceived restlessness, identify the need for resources, and also promote cooperation between the systems they live in.

### Integration of perspectives

Reflecting on the restless child as possible victim of maltreatment, created an interesting dynamic in the group: a distinct silence was followed by reflections on whether we *really understand*. Interestingly, being released from complexity, confusion, guilt, and responsibility is held to be an important function of the ADHD diagnosis (Neufeld & Foy, 2006). If ADHD serves this function, that adults get the benefit of avoiding children's, well as their own, pain and confusion, should it still be considered a useful concept for practice and research? How could this potentially destructive aspect of diagnostic practice be amended or avoided?

One possible strategy could be to systematically integrate a *child perspective* in both practice and research (Sherr, Skar, Clucas, Von Tetzchner, & Hundeide, 2013; Sommer et al., 2010). Child perspectives direct adults’ attention towards an understanding of children's perceptions, experiences, and actions in the world, and can prevent “difficult” children from being expelled from the zone of intimacy where empathic care takes place (Sommer et al., 2010). Through her research, Olsvold (2012) shows how the relational dynamic and focus for communication might change as the ADHD diagnosis “enters,” and that this change undermines a child perspective and obscures the child's own perspectives.

A second strategy could be to integrate a child maltreatment perspective in both research and practice. A developmental perspective on complex traumatization (Braarud & Nordanger, 2011) is one example of such integration, and can help both researchers, practitioners and parents to understand restlessness in terms of regulation. Roughly speaking, a regulation perspective can indicate that the restless child is bored and expresses a need for stimulation or that the child is overstimulated, scared, or feels threatened and needs help to calm down and/or feel safe. An unmet need for regulation over time, like neglect or abuse, can disturb the child's development and create both internal and external restlessness.

The very popularity of the ADHD diagnosis has been explained with its potential to release adults from responsibility, confusion, and shame (Neufeld & Foy, 2006). This could explain the finding that parents and professionals need to be reminded about the relational responsibility and ecological complexity. It might also explain why research on ADHD and on child maltreatment is poorly integrated despite evidence that suggests a strong connection between the two (Fuller-Thomson et al., 2014). Currently, being traumatized and having ADHD are treated as a question of differential diagnosis, and some children are given both. In practice, however, ADHD is the most widely used diagnosis, even though child maltreatment is estimated to be a bigger public health problem both globally (Stoltenborgh et al., 2015) and in the Nordic countries (Kloppen et al., 2014).

Child maltreatment is an important and poorly integrated aspect. However, not all restlessness stems from experiences of complex traumatization. As the participants pointed out, many parents need to use fewer critical and negative strategies when communicating with their children. Many children also overhear parents’ arguments or sense relational tensions without having the means to understand or handle these. One participant's comment about the shared responsibility to remind parents of their position and responsibility might indicate that there exists a culture of avoidance in the adult population, and that ADHD serves to facilitate this.

## Summary and conclusion

Our findings suggest that adults from one local community in western Norway that are involved in children's everyday life describe children's restless behaviors as an everyday challenge but seem to resist the individual and pathology focused explanations provided by a biomedical perspective. Participants resisted the medicalization of children's restlessness by sharing everyday reflections that outlined a need for more ecologically valid understandings, a new sense of solidarity in the face of children's problems, and increased sustainability of practice. Discussions regarding child maltreatment lead to a deep and genuine wish to *understand* children better. The findings from our study correspond to findings from critical research on ADHD where basic questions about the validity of the ADHD diagnosis and the sustainability of medical treatment of symptoms are heavily debated.

Our findings point to possible implications on several ecological levels. At a micro level, our findings point to the need for more awareness about the relational nature of restlessness which in turn might point to the need for resources to better handle children's restlessness and creativity in everyday settings. At a meso level, our findings point to a need for increased and improved cooperation between institutions in the local community. At a macro level, our findings indicate a need for a more reflexive approach to children's restlessness, which again could act to increase the validity of our understandings, and facilitate solidarity and sustainability in the actions we take when faced with children's restlessness. Rather than being neutral observers or helpers, adults co-create children's problems through their interactions with children and through their interpretations of children. As co-creators, adults share the responsibility to resolve children's problems. Through becoming aware of our role and responsibility as co-creators, we can facilitate interpretations of children's restlessness that better correspond to their own perspectives and contribute to sustainable solutions in their life-worlds.

## References

[CIT0001] Ackerman P. T, Newton J. E. O, McPherson W. B, Jones J. G, Dykman R. A (1998). Prevalence of post traumatic stress disorder and other psychiatric diagnoses in three groups of abused children (sexual, physical, and both). Child Abuse & Neglect.

[CIT0002] Alvesson M, Sköldberg K (2000). Reflexive methodology.

[CIT0003] American Psychatric Association (2013). Diagnostic and statistical manual of mental disorders.

[CIT0007] Binder P.-E, Holgersen H, Moltu C (2012). Staying close and reflexive: An explorative and reflexive approach to qualitative research on psychotherapy. Nordic Psychology.

[CIT0008] Braun V, Clarke V (2012). Using thematic analysis in psychology. Qualitative Research in Psychology.

[CIT0009] Bronfenbrenner U (1977). Toward an experimental ecology of human development. American Psychologist.

[CIT0010] Bronfenbrenner U (1979). The ecology of human development: Experiments by nature and design.

[CIT0013] Braarud H. C, Nordanger D. Ø (2011). Kompleks traumatisering hos barn: En utviklingspsykologisk forståelse. Tidsskrift for Norsk Psykologforening.

[CIT0014] Bøe T (2013). Socioeconomic status and mental health in children and adolescents.

[CIT0015] Bøe T, Øverland S, Lundervold A, Hysing M (2012). Socioeconomic status and children's mental health: Results from the Bergen Child Study. Social Psychiatry and Psychiatric Epidemiology.

[CIT0016] Charach A, Yeung E, Volpe T, Goodale R, Dos Reis S (2014). Exploring stimulant treatment in ADHD: Narratives of young adolescents and their parents. BMC Psychiatry.

[CIT0017] Dalton J. H, Elias M. J, Wandersman A (2007). Community psychology. Linking individuals and communities.

[CIT0020] Foros P. B, Vetlesen A. J (2012). Angsten for oppdragelse: et samfunnsetisk perspektiv på dannelse.

[CIT0021] Fuller-Thomson E, Mehta R, Valeo A (2014). Establishing a link between attention deficit disorder/attention deficit hyperactivity disorder and childhood physical abuse. Journal of Aggression, Maltreatment & Trauma.

[CIT0023] Helle-Valle A (2014). How do we understand restlessness? A critique of the biopsychosocial model and ADHD as the dominating perspective in current understanding and treatment. Voices: A World Forum for Music Therapy.

[CIT0024] Heron J, Reason P, Reason P, Bradbury H (2001). The practice of co-operative inquiry: Research ‘with’ rather than ‘on’ people. The handbook of action research.

[CIT0025] Hummelvoll J. K (2006). Action oriented research cooperation—Theoretical grounds and practical implications. Norsk Tidsskrift for Sykepleieforskning.

[CIT0026] Kloppen K, Mæhle M, Kvello Ø, Haugland S, Breivik K (2014). Prevalence of intrafamilial child maltreatment in the Nordic countries: A review. Child Abuse Review.

[CIT0028] Lillemoen P. K. S, Kjosavik S. R, Hunskår S, Ruths S (2012). Prescriptions of drugs against AD/HD 2004–08. Tidsskrift for den Norske Legeforening.

[CIT0029] Luman M, Tripp G, Scheres A (2010). Review: Identifying the neurobiology of altered reinforcement sensitivity in ADHD: A review and research agenda. Neuroscience and Biobehavioural Reviews.

[CIT0030] Moffitt T. E, Houts R, Asherson P, Belsky D. W, Corcoran D. L, Hammerle M (2015). Is adult ADHD a childhood-onset neurodevelopmental disorder? Evidence from a four-decade longitudinal cohort study. American Journal of Psychiatry.

[CIT0031] Molina B. S. G, Hinshaw S. P, Swanson J. M, Arnold L.-E, Vitiello B, Jensen P. S (2009). The MTA at 8 years: Prospective follow-up of children treated for combine-type ADHD in a multisite study. Journal of the American Academy of Child and Adolescent Psychiatry.

[CIT0032] Navarez D, Panksepp J, Schore A. N, Gleason T (2013). Evolution, early experience and human development: From research to practice and policy.

[CIT0033] Neufeld P, Foy M (2006). Historical reflections on the ascendancy of ADHD in North America, c. 1980–c. 2005. British Journal of Educational Studies.

[CIT0034] Olsvold A (2012). Når “ADHD” kommer inn døren: En psykososial undersøkelse av barns, mødres og fedres forståelse og opplevelse av ADHD-diagnose og -medisinering.

[CIT0036] Panksepp J (1998). Attention deficit hyperactivity disorders, psychostimulants, and intolerance of childhood playfulness: A tragedy in the making?. Current Directions in Psychological Science.

[CIT0037] Polanczyk G, De Lima M. S, Horta B. L, Biederman J, Rohde L. A (2007). The Worldwide prevalence of ADHD: A systematic review and metaregression analysis. American Journal of Psychiatry.

[CIT0038] Rowland A. S, Lesesne C. A, Abramowitz A. J (2002). The epidemiology of attention-deficit/hyperactivity disorder (ADHD): A public health view. Mental Retardation and Developmental Disabilities Research Reviews.

[CIT0039] Russell G, Ford T, Rosenberg R, Kelly S (2014). The association of attention deficit hyperactivity disorder with socioeconomic disadvantage: Alternative explanations and evidence. Journal of Child Psychology and Psychiatry.

[CIT0040] Sherr L, Skar A.-M. S, Clucas C, Von Tetzchner S, Hundeide K (2013). Evaluation of the International Child Development Programme (ICDP) as a community-wide parenting programme. European Journal of Developmental Psychology.

[CIT0041] Singh I (2003). Boys will be boys: Fathers’ perspectives on ADHD symptoms, diagnosis, and drug treatment. Harvard Review of Psychiatry.

[CIT0042] Singh I (2004). Doing their jobs: Mothering with Ritalin in a culture of mother-blame. Social Science & Medicine.

[CIT0043] Singh I (2013). Not robots: Children's perspectives on authenticity, moral agency and stimulant drug treatments. Journal of Medical Ethics.

[CIT0044] Singh I, Filipe A, Bard I, Bergey M, Baker L (2013). Globalization and cognitive enhancement: Emerging social and ethical challenges for ADHD clinicians. Current Psychiatry Report.

[CIT0045] Sommer D, Samuelsson I. P, Hundeide K (2010). Child perspectives and children's perspectives in theory and practice (Vol. 2).

[CIT0046] Stjernø S (2004). Solidarity in Europe: The history of an idea.

[CIT0047] Stoltenborgh M, Bakermans-Kranenburg M. J, Alink L. R, Van Ijzendoorn M. H (2015). The prevalence of child maltreatment across the globe: Review of a series of meta-analyses. Child Abuse Review.

[CIT0048] Thorkildsen I. M (2015). Du ser det ikke før du tror det. Et kampskrift for barns rettigheter.

[CIT0049] Timimi S, Leo J (2009). Rethinking ADHD, from brain to culture.

[CIT0050] Tripp G, Wickens J. R (2009). Neurobiology of ADHD. Neuropharmacology.

[CIT0051] Ullebø A. K (2010). Epidemiology of ADHD. Screening, prevalence and phenomenology of the attention deficit/hyperactivity disorder phenotype.

[CIT0052] United Nations (1989). Convention on the rights of the child: Adopted and opened for signature, ratification and accession by General Assembly resolution 44/25 of 20 November 1989 entry into force 2 September 1990, in accordance with article 49.

[CIT0053] Van der Kolk B. A (2005). Developmental trauma disorder: Towards a rational diagnosis for chronically traumatized children. Psychiatric Annals.

